# The positive effects of Xueshuan Xinmai tablets on brain functional connectivity in acute ischemic stroke: a placebo controlled randomized trial

**DOI:** 10.1038/s41598-017-15456-9

**Published:** 2017-11-10

**Authors:** Dongfeng Wei, Daojun Xie, He Li, Yaojing Chen, Di Qi, Yujiao Wang, Yangjun Zhang, Kewei Chen, Chuanfu Li, Zhanjun Zhang

**Affiliations:** 1 Institute of Basic Research in Clinical Medicine, China Academy of Chinese Medical Sciences, Beijing, 100700 P.R. China; 2 The First Affiliated Hospital of Anhui University of traditional Chinese Medicine, Hefei, 230031 P.R. China; 3 State Key Laboratory of Cognitive Neuroscience and Learning & IDG/McGovern Institute for Brain Research, Beijing Normal University, Beijing, 100875 P.R. China; 4 BABRI Centre, Beijing Normal University, Beijing, 100875 P.R. China; 5 Graduate School of Anhui University of traditional Chinese Medicine, Hefei, 230038 P.R. China; 6 Banner Alzheimer’s Institute, Phoenix, Arizona 85006 USA

## Abstract

Through a placebo controlled randomized study, the purpose of this report was to investigate the effects of Xueshuan Xinmai tablets (XXMT) on neurologic deficits, quality of life and brain functional connectivity in acute ischemic stroke patients and to explore the mechanism of action of XXMT. In total, 44 acute ischemic stroke patients were randomly divided to the XXMT treatment group (n = 22) or the placebo group (n = 22) in a 2-week trial. Before and after the treatment, the neurological assessment and functional magnetic resonance imaging examinations were carried out. Compared to the placebo group, the scores of the National Institutes of Health Stroke Scale (NIHSS) and Stroke-Specific Quality of Life Scale (SSQOL) significantly improved in the treatment group. In addition, XXMT-treated patients demonstrated significantly enhanced functional connectivity within the default mode, frontal-parietal, and motor control networks. Furthermore, the changed connectivity in the left precuneus was positively correlated to the improvement of NIHSS and SSQOL scores. The present study indicated that XXMT treatment significantly improved the neurologic deficit and quality of life of acute ischemic stroke patients and that the therapeutic effect may be based on the modulation of XXMT on the functional connectivity of brain networks.

## Introduction

Stroke, also known as cerebrovascular disease, is the second most common cause of death and the leading cause of acquired long-term disability in adults worldwide^1^. Ischemic stroke, which is caused by large cerebral artery occlusion, is the primary pathological type of stroke and an important contributor to vascular dementia and cognitive impairment^2^. Therefore, the early diagnosis and intervention at the acute stage of ischemic stroke are vital in the prevention of the stroke recurrence and subsequent dementia. These early measures are also considered critical and preventive part of therapies for vascular dementia^3^.

Currently, common clinical practice in treating patients with an acute ischemic stroke involves fibrinolytic therapy (administration of recombinant tissue-type plasminogen activator, rt-PA) and antiplatelet agents (such as aspirin)^4,5^. However, rt-PA treatment is more effective only if given within approximately 3–4.5 hours of symptoms onset^6,7^. Unfortunately, merely a small percentage of patients can be treated within this time frame in China^8,9^. Meanwhile, several studies have reported that antiplatelet therapy did not significantly reduce the risk of recurrent stroke as expected, but instead it considerably increased the risk of bleeding and death^10,11^. The limitations of these treatments, likely reflecting the multiple mechanisms involved in the pathogenesis of ischemic strokes, thereby prompted the desire to explore other treatment regimens.

Traditional Chinese medicine (TCM) provides an alternative therapy for ischemic stroke and has been widely used in stroke treatment in China^12,13^. Xueshuan Xinmai tablets (XXMT) is a traditional Chinese patented medicine that is commonly used in clinical practice and that has been approved for ischemic stroke by the China Food and Drug Administration in 2003 (Z20030145). XXMT is composed of more than a dozen Chinese herbs and is based on the TCM theory of these raw materials. Treatment efficacy with this medicine has been further refined using modern advanced technology^14^. *Ligusticum wallichii* (chuanxiong in Chinese) and *Salvia miltiorrhiza* (danshen in Chinese) are two major components of XXMT. As a compound medicine of Chinese herbs, XXMT is functionally characterized as benefiting qi via activating blood circulation (Yiqi Huoxue in Chinese) and shows a better therapeutic effect on acute ischemic stroke patients. However, as with any other traditional Chinese medicine compound, XXMT includes a variety of active ingredients, thus it is difficult to explain the therapeutic mechanism of action.

Resting-state functional magnetic resonance imaging (rs-fMRI) is a promising and noninvasive neuroimaging technique to measure spontaneous and intrinsic brain activity under no external input or performance demand^15^. It has been recently used to investigate the functional changes in brain networks and recovery after stroke^16–21^. These resting-state fMRI studies on ischemic stroke have confirmed the disruption or reorganization of intrinsic connectivity not only between cortical areas around the ischemic lesion but also in widely distributed neural networks, including the default mode network (DMN)^16,20^, the fronto-parietal network (FP)^18^ and the motor network^17,19^. Interestingly, the disrupted neural networks in patients with strokes always exhibited restoration accompanied with the recovery of neurologic deficit and quality of life after treatment, a fact that is credited to neural plasticity^17,22^.

In the present study, a placebo-controlled clinical trial was performed to assess the therapeutic effect of XXMT on acute ischemic stroke patients using both stroke scales and rs-fMRI technique measures to define the outcomes. We hypothesized that 1) the neurologic deficit and quality of life of patients with acute ischemic stroke can be improved after two weeks of treatment with XXMT, and that 2) the therapeutic effect of XXMT is associated with its modulation on the resting-state functional connectivity of the neural networks.

## Results

### Demographics, neurological testing and blood biomarker detection

There were no differences in age (*P* = 0.085), gender (*P* = 0.899) or education (*P* = 0.566) between the placebo and the treatment groups. The group differences in the scores of the National Institute of Health Stroke Scale (NIHSS), the Barthel index, the Modified Rankin Scale (mRS) and the Stroke-specific quality of life scale (SSQOL) did not reach statistical significance before drug intervention (Table 1). The distribution of the ischemic lesions of the two groups are presented in Supplementary Table S1. The ischemic lesions mainly include the basal ganglia, corona radiata, and brainstem, however, some are located in the thalamus, caudate nucleus, external capsule, temporal lobe, parietal lobe, frontal lobe, and occipital lobe. The location of the infarct lesion had no significant difference between the two groups. The T1- and T2-weighted magnetic resonance imaging (MRI) of an acute ischemic stroke patient with infarct lesion were shown in Supplementary Fig. S1.

**Table 1:** Demographic information.

	Placebo group (n = 20)	Treatment group (n = 22)	*T*/*χ* ^2^ value	*P* value
Age (y)	59.05(9.58)	64.63(10.80)	−1.765	0.085
Gender (M/F)	14:6	15:7	0.016	0.899
Education (y)	8.60(5.16)	7.73(4.60)	0.579	0.566
NIHSS	5.85(3.28)	6.64(3.09)	0.638	0.429
Barthel	68.50(26.01)	66.81(26.07)	0.048	0.828
mRS	2.80(1.28)	3.18(1.18)	0.922	0.343
SSQOL	147.35(41.47)	125.05(43.63)	2.011	0.165

Values are mean(SD) or number of patients. NIHSS: National Institute of Health Stroke Scale; mRS: Modified Rankin Scale; SSQOL: Stroke-Specific Quality of Life Scale.

When repeated-ANOVA was conducted with age, sex, education and infarct volume as the covariates, we found that the interaction of group and time was significant on the NIHSS score (F = 5.402, *P* = 0.026), SSQOL score (F = 9.755, *P* = 0.003) and the hematocrit value (F = 4.601, *P* = 0.039) (Table 2). This finding indicates that drug therapy plays a positive role in improving general physical function and blood viscosity in patients with acute ischemic stroke. Infarct volume change can reflect the degree of rehabilitation of the acute ischemic stroke patients. Although the interaction was not significant, the infarct volume of the drug treatment group had a downward trend after XXMT treatment, while the infarct volume of the placebo group had an upward trend.

**Table 2:** Common stroke scale test and blood biomarker detection results.

	Placebo group (n = 20)		Treatment group (n = 22)		Main effect of time		Main effect of group		Interaction	
	Baseline	2 weeks later	Baseline	2 weeks later	F value	*P* value	F value	*P* value	F value	*P* value
NIHSS	5.85(3.28)	3.65(2.80)	6.64(3.09)	3.23(2.41)	0.079	0.780	0.079	0.780	5.402	0.026^*^
Barthel	68.50(26.01)	80.75(19.49)	66.82(26.07)	67.62(25.74)	0.563	0.458	0.026	0.872	1.661	0.205
mRS	2.80(1.28)	2.00(1.33)	3.18(1.18)	1.73(1.32)	2.069	0.159	0.043	0.837	4.038	0.052
SSQOL	147.35(41.47)	189.40(37.97)	125.05(43.63)	204.05(44.27)	1.466	0.234	0.024	0.877	9.755	0.003^**^
Infarct volume	43280.70 (26358.07)	48388.95 (29839.52)	41005.36 (20465.21)	38572.29 (20386.29)	1.470	0.212	0.039	0.845	1.365	0.250
Fibrinogen	2.83(0.50)	2.75(0.52)	3.26(0.99)	9.26(1.12)	0.116	0.736	2.506	0.122	0.042	0.838
Hematocrit	39.09(4.72)	39.54(4.53)	39.70(4.41)	38.53(3.54)	3.978	0.054	0.001	0.980	4.601	0.039^*^
Total cholesterol	5.00(1.39)	4.67(0.99)	4.64(1.22)	4.34(0.98)	2.222	0.145	2.205	0.333	0.02	0.888
Triglyceride	1.81(1.99)	1.92(1.97)	1.91(1.00)	1.85(0.95)	0.013	0.909	0.131	0.719	0.948	0.336
Low-density lipoprotein	3.00(0.90)	2.77(0.67)	2.76(0.96)	2.54(0.68)	0.900	0.349	1.572	0.218	0.005	0.942
High-density lipoprotein	1.41(0.57)	1.22(0.25)	1.10(0.20)	1.17(0.27)	2.406	0.129	4.081	0.051	2.056	0.160

Values are mean (SD) or number of patients. NIHSS: National Institute of Health Stroke Scale; mRS: Modified Rankin Scale; SSQOL: Stroke-Specific Quality of Life Scale; ^*^ *P* < 0.05, ^**^ *P* < 0.01.

### Independent component analysis (ICA) based functional connectivity of the neural networks

**Figure 1:**
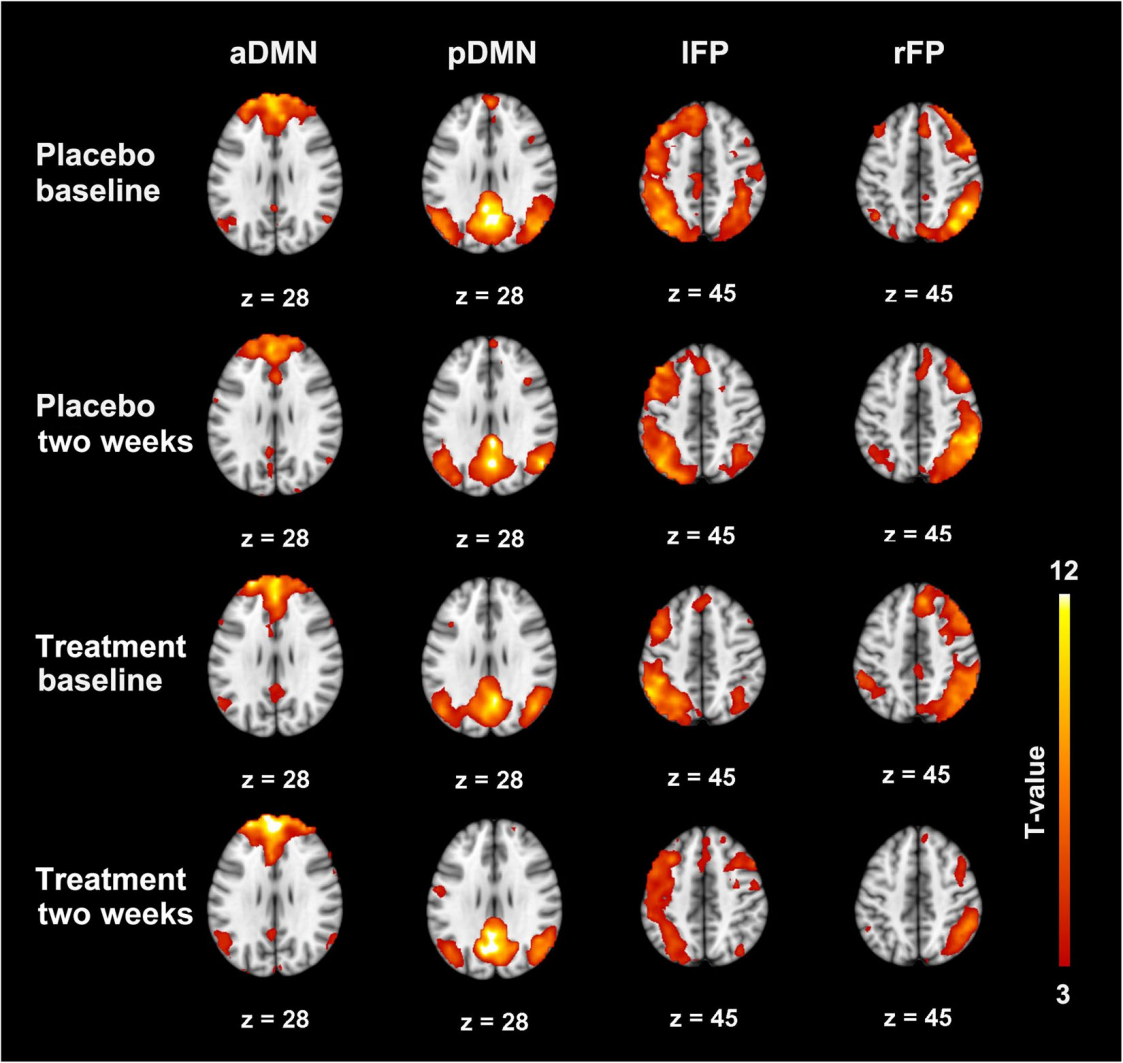
Pattern of four networks in the XXMT treatment group and the placebo group was obtained by applying spatial ICA and one-sample t-test to the resting-state fMRI data. Abbreviation: aDMN, the anterior default mode network; pDMN, posterior default mode network; lFP, left frontal parietal network; and rFP, right frontal parietal network.

By applying spatial ICA to the resting-state fMRI data, we obtained four brain networks in the two groups of patients, as shown in Fig. 1. Specifically, the anterior DMN (aDMN) included the medial prefrontal cortex. The posterior DMN (pDMN) included the precuneus, posterior cingulate, and both sides of the inferior parietal lobule. The left FP (lFP) and right FP (rFP) included the left frontal and parietal lobes and the right frontal and parietal lobes, respectively.

**Figure 2:**
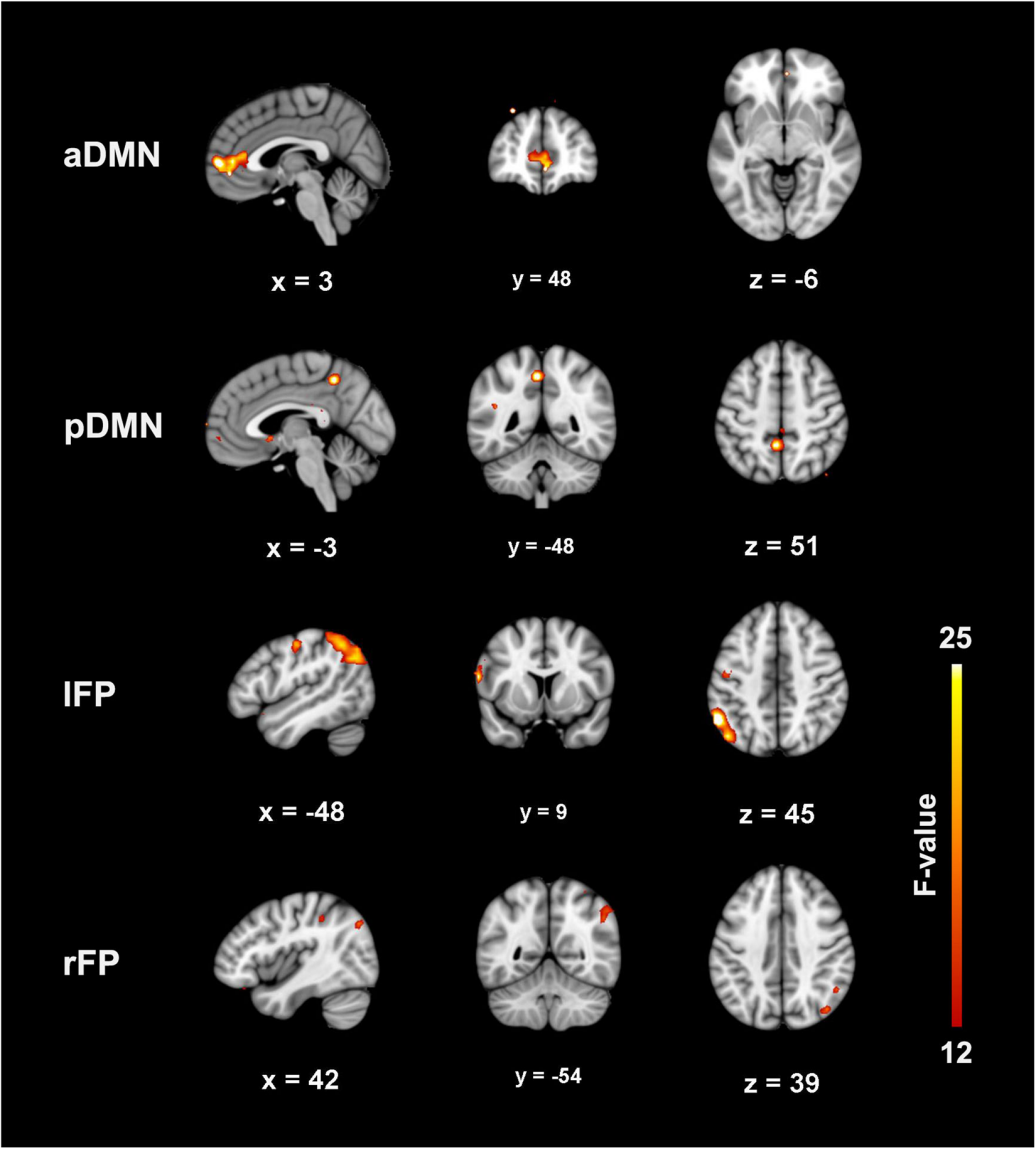
The interaction of group and time on the four networks was shown (*P* < 0.001, uncorrected). Abbreviation: aDMN, anterior default mode network; pDMN, posterior default mode network; lFP, left frontal parietal network; and rFP, right frontal parietal network.

Full-factorial analyses revealed that the functional connectivity of the right medial prefrontal cortex (x = 3, y = 48, z = −6) in the aDMN, the left precuneus (x = −3, y = −48, z = 51) in the pDMN, as well as the left opercular inferior frontal cortex (x = −60, y = 9, z = 18) and the left postcentral cortex (x = −48, y = −15, z = 48) in the lFP increased in the treatment group compared with the placebo group (*P* < 0.001, uncorrected). Nevertheless, the decreased functional connectivity of the left inferior parietal lobe (x = −54, y = −54, z = 45) in the lFP as well as the right inferior parietal lobe (x = 51, y = −54, z = 39) and the right angular gyrus (x = 42, y = −72, z = 39) in the rFP was found in the treatment group compared with the placebo group (*P* < 0.001, uncorrected) (Fig. 2, Table 3).

**Table 3:** The group by time interaction of four network.

Network	Clusters	Voxels	Peak coordinate			Brain regions
			x	y	z	
**aDMN**						
	1	309	3	48	−6	Right medial prefrontal cortex
**pDMN**						
	1	21	−3	−48	51	Left precuneus
**lFP**						
	1	88	−60	9	18	Left opercular inferior frontal cortex
	2	382	−54	−54	45	Left inferior parietal gyrus
	3	29	−48	−15	48	Left postcentral
**rFP**						
	1	23	42	−72	39	Right angular
	2	25	51	−54	39	Right inferior parietal gyrus

aDMN: anterior default mode network; pDMN: posterior default mode network; lFP: left frontal-parietal network; rFP: right frontal-parietal network.

### Functional connectivity of motor-related regions of interest (ROIs)

**Figure 3:**
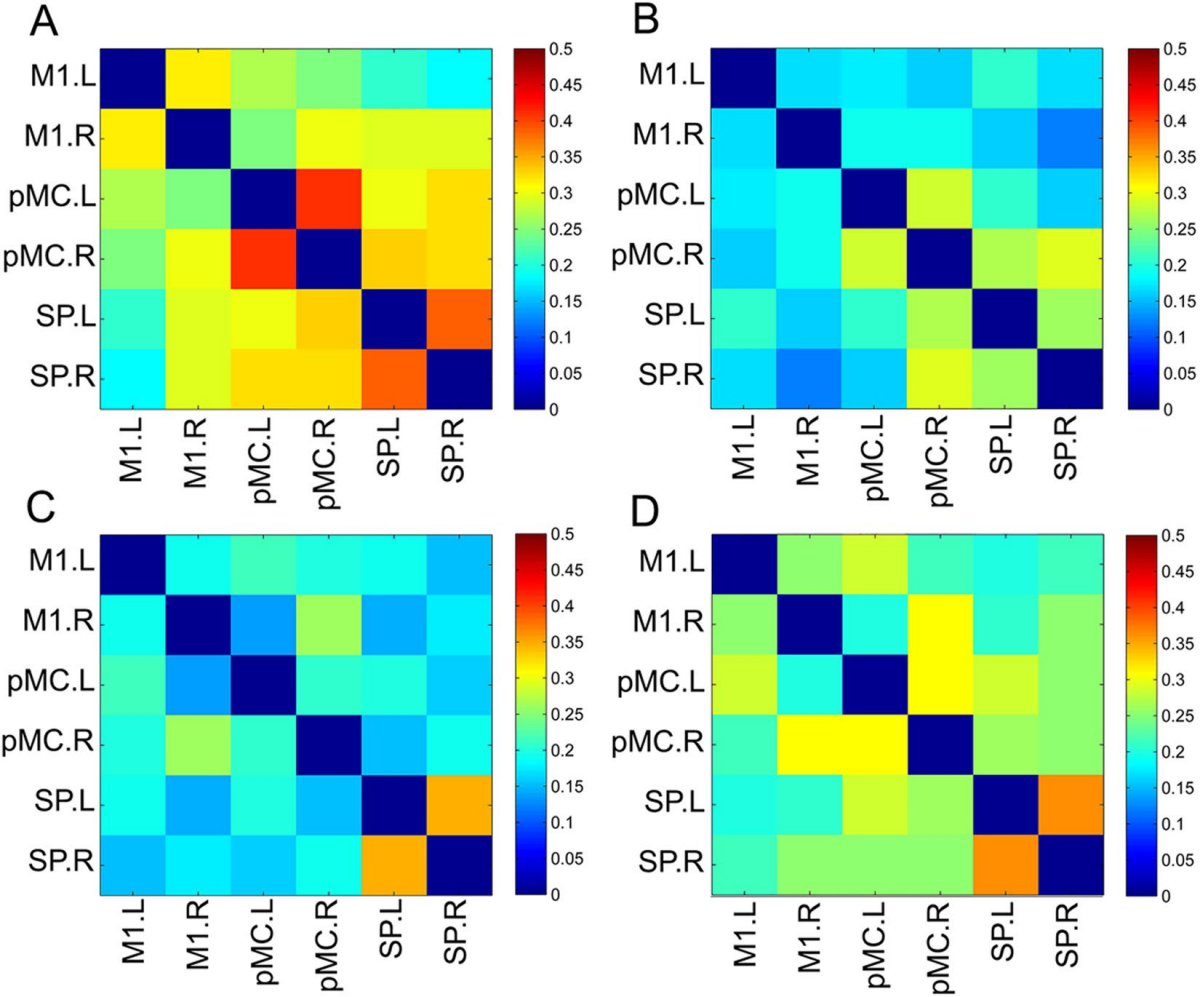
The connection matrices of the six motor-related ROIs in the XXMT treatment group and the placebo group at baseline and at the second visit (2 weeks later) was shown (FDR correction, *P < *0.05). (**A**) placebo-baseline; (**B**) placebo-2 weeks later; (**C**) treatment-baseline; D. treatment-2 weeks later. Abbreviation: M1.L, left primary motor cortex; M1.R, right primary motor cortex; pMC.L, left premotor cortex; pMC.R, right premotor cortex; SP.L, left superior parietal cortex; SP.R, right superior parietal cortex.

The connection matrices of the six motor-related ROIs in the two groups at two time points are displayed in Fig. 3. In the network constructed by 6 ROIs, the left primary motor cortex-right primary motor cortex connectivity (F = 7.117, *P* = 0.011), right primary motor cortex-right superior parietal cortex connectivity (F = 10.762, *P* = 0.002), left premotor cortex-right premotor cortex connectivity (F = 8.066, *P* = 0.007), and left premotor cortex-right superior parietal cortex connectivity (F = 9.624, *P* = 0.004) increased in the treatment group compared with the placebo group (*P* < 0.05, FDR corrected).

### The correlations between functional connectivity and neurological scores

**Figure 4:**
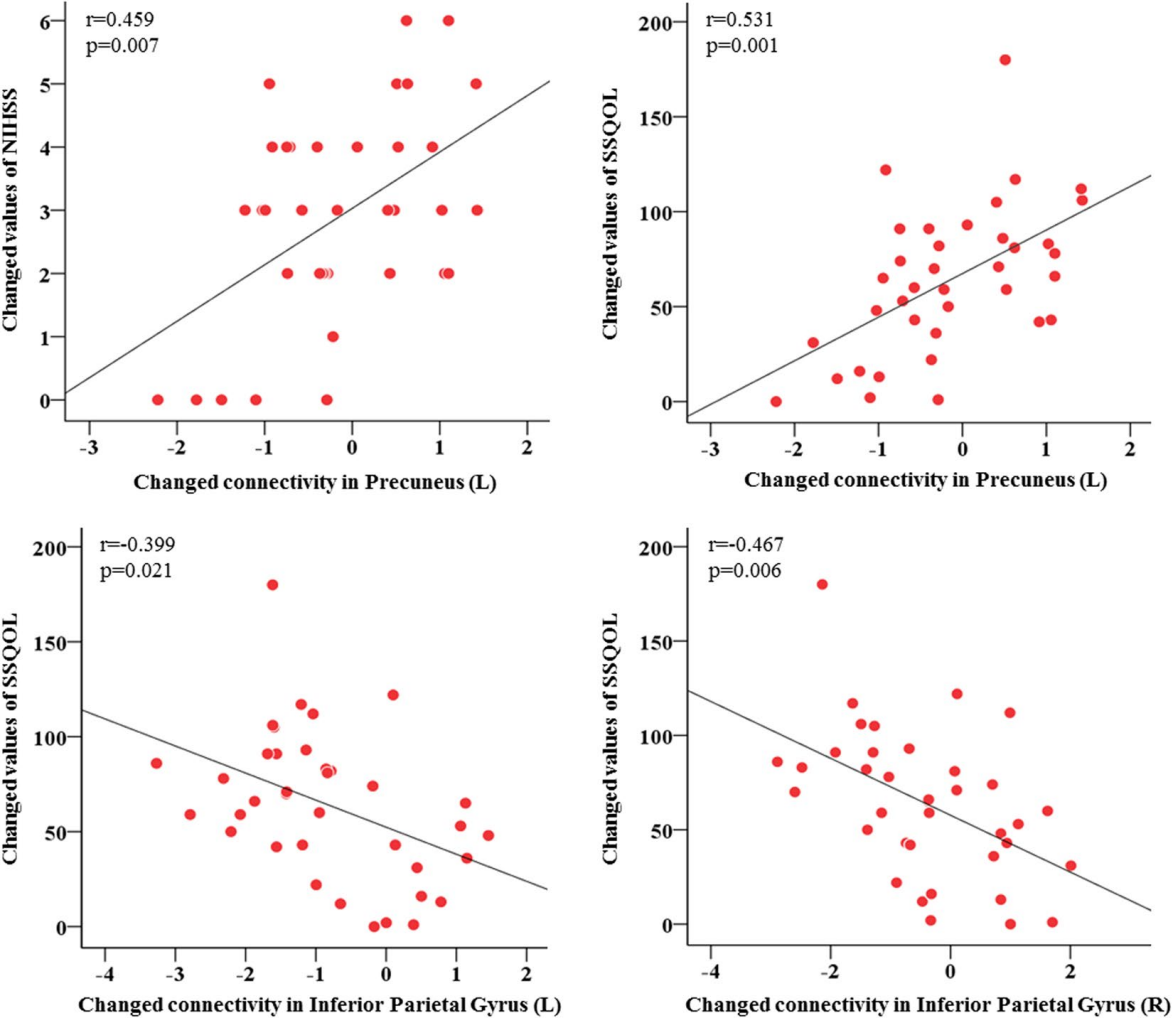
Correlations between changed connectivity of brain regions and changed values of common stroke scale scores in acute ischemic stroke patients.

The correlations between changed connectivity of ROIs, which had a significant interaction effect between time and group, and changed values of common stroke scale scores was examined in acute ischemic stroke. The changed values of NIHSS score was significantly related with changed connectivity in the precuneus (L) (r = 0.459, *P* = 0.007). The changed values of SSQOL score was significantly related with changed connectivity in the precuneus (L) (r = 0.531, *P* = 0.001), inferior parietal gyrus (L) (r = −0.399, *P* = 0.021) and inferior parietal gyrus (R) (r = −0.467, *P* = 0.006) (Fig. 4 and Supplementary Table S2).

## Discussion

Our present study explored the impact of two-week XXMT treatment on neurologic deficit, quality of life and resting state brain network connectivity in acute ischemic stroke patients. Our results showed that XXMT treatment significantly improved neurologic deficit, quality of life and blood viscosity but not infarct volume. In our subsequent analysis of the fMRI data, we found that XXMT could improve brain resting state functional connectivity of the DMN network, mainly in the right medial frontal cortex and the left precuneus. Our results also showed that XXMT could increase the connectivity of the left opercular inferior frontal cortex and the left postcentral cortex within the frontal-parietal network, and a number of connections in the motor control network. Moreover, in the treatment group, we observed a significant association between NIHSS/SSQOL and the functional connectivity at the core regions in the aDMN, pDMN, lFP and rFP, where a significant interaction effect between time and group was observed.

TCM has now been receiving more and more attention as an alternative treatment for various diseases, including the prevention and treatment of ischemic stroke. TCM has evolved into a complete medical system that has diagnosed, treated, and prevented illness and takes a holistic view of the human system over the past thousands of years. To some degree, this holistic intervention model could be beneficial and complementary to the single-target intervention model of modern medicine. Interestingly, many studies have confirmed the beneficial effects of TCM as a treatment for acute ischemic stroke^23–25^. XXMT has been widely used in the TCM clinical practice for treating ischemic stroke, cerebral thrombosis and coronary heart disease and is effective because it enhances blood circulation and removes blood stasis^26,27^. Previous studies have demonstrated that tetramethylpyrazine (TMP), the main extract of ligusticum wallichii, is a biologically active alkaloid that promotes vasodilatation, inhibits platelet aggregation and exhibits significant antioxidant effects^28,29^. Similarly, tanshinone IIA (Tan IIA), the main extract of salvia miltiorrhiza, is a lipid-soluble active compound that possesses potential anti-inflammatory, anti-oxidant, anti-apoptotic and neuroprotective properties^30,31^. Synergistically, the combination of Tan IIA and TMP is a widely applied therapy against ischemic stroke in TCM^30^. These biochemical findings provided support for our results.

There are many differences between this study and our previous work (Int J Clin Exp Med 2015;8(5):7507–7516) on the study purpose, patient diagnosis criteria, demographics, treatment period, clinic behavior assessmentscale, brain functional imaging analysis method and study results. The present study aims to investigate the effects of XXMT on neurologic deficit, quality of life and brain functional connectivity in acute ischemic stroke patients and to explore the mechanism of action of XXMT through a placebo controlled randomized study. The patients were recruited from the First Affiliated Hospital of AnHui College of Traditional Chinese Medicine from January 1st, 2012 to December 29st, 2012. The patients meet acute ischemic stroke diagnosis criteria and received 2 weeks XXMT treatment. The study results indicated that XXMT treatment significantly improved the neurologic deficit and quality of life of acute ischemic stroke patients and that the therapeutic effect may be based on the modulation of XXMT on the functional connectivity of brain networks including default mode network, frontal-parietal network, motor control network. The purpose of our previous work “The therapeutic effect of Xueshuan Xinmai tablets on memory injury and brain activity in post-stroke patients: a pilot placebo controlled fMRI study” was to explore the effects of XXMT for the treatment of cognitive function, brain region activation in the rehabilitation period of ischemic stroke patients. The patients were recruited from eight communities from January 1st, 2012 to June 1st, 2013. The patients meet chronic ischemic stroke diagnosis criteria in the rehabilitation period (between 15 days and 6 months after the onset of symptoms) and received 3 months XXMT treatment. The results showed that XXMT treatment has a favorable mediation on episodic memory, consequently suppresses the activation of the cingulate gyrus in the rehabilitation period of ischemic stroke patients.

NIHSS is a widely used test in strokes and is composed of language, cognitive ability, visual field defect, sensation, and reflection^32^. In this study, we observed a significant decrease in the NIHSS score in the treatment group compared with the placebo group. As the second index we used in this study, SSQOL includes the physical function, social participation, and psychological and subjective feelings among others^33^. We also observed a significant increase of SSQOL score in the treatment group compared with the placebo group. Both indices pointed to the improvements that XXMT had on neurologic function deficits and quality of life in acute ischemic stroke patients. On the other hand, no significant effect on reducing infarct volume was observed over the two-week treatment, suggesting that the effects of XXMT are primarily functional in acute ischemic stroke. Another possibility is that the sample size is too small to detect the volumetric change of the infarct lesion.

Regarding the brain’s plasticity, the brain may compensate for functional impairment through reorganization of the neural networks^34,35^. Previous brain network studies in stroke patients mainly focused on motor-related networks and DMN^21,36^. Park *et al*. found that the stroke patients showed a markedly decreased DMN functional connectivity in the posterior cingulate cortex (PCC), precuneus, and medial frontal cortex (MFG) one month after stroke compared with a group of healthy controls^20^. In the present study, ICA, which is a computational method for separating a multivariate signals into independent non-Gaussian signals, was used to identify DMN and FPN functional connectivity analysis. Subsequent network analysis revealed that XXMT treatment significantly increased DMN functional connectivity in the right medial prefrontal cortex and left precuneus, and also increased lFP functional connectivity in the left opercular inferior frontal cortex and left postcentral cortex in acute ischemic stroke patients. This finding demonstrated that XXMT treatment can improve cognitive impairment by increasing functional connectivity in these brain regions that are affected in acute ischemic stroke. Furthermore, our findings suggest that functional connectivity evaluation of these brain regions can serve as a potential marker to assess the clinical effects of treatment on ischemic stroke patients.

In this study, six ROIs, which are related to motor control and motor execution function, were included in pair-wise correlations in motor networks functional connectivity analysis, and a symmetric correlation matrix for each subject was obtained. XXMT treatment significantly increased functional connectivity within the motor network: between the left primary motor cortex and right primary motor cortex, between the right primary motor cortex and superior parietal cortex, between the left premotor cortex and right premotor cortex, and between the left premotor cortex and right superior parietal cortex. These brain regions all belong to the motor network, playing an important role in the recovery of motor function in acute ischemic stroke patients^37,38^. While Inman *et al*. showed that functional connectivity of the brain regions in the motor control network, including the primary motor cortex and superior parietal cortex, significantly decreased in stroke patients^37^, we demonstrated that XXMT treatment can improve neurological function deficits through increasing functional connectivity of these brain regions within the motor network in acute ischemic stroke patients.

Our analysis on the relationship between the network functional connectivity and the stroke scale scores, NIHSS and SSQOL, indicated that they are significantly associated. Such an association contributes to our understanding of the neural integration mechanisms that are affected by XXMT in acute ischemic stroke. Furthermore, these brain regions and functional connectivity should be further analyzed as a curative effect of the potential treatment for ischemic stroke.

Several limitations exist in the current study and should be addressed in the future. The limitations include a small sample size, a short duration and a need for evaluation of multiple domains of cognition. Future longitudinal studies are needed and would be essential to confirm our findings.

In summary, the present study demonstrated that XXMT treatment significantly improves the neurologic deficit and quality of life of acute ischemic stroke patients over two weeks and that this therapeutic effect may be related to the modulation of XXMT on the functional connectivity with brain networks. These results provide some insights into future treatments for acute ischemic stroke patients. Subsequent studies with a larger sample size and longer treatment duration should explore the curative effect and underlying mechanisms of action of compound TCM treatment on acute ischemic stroke patients.

## Methods

### Study Design and Participants

**Figure 5:**
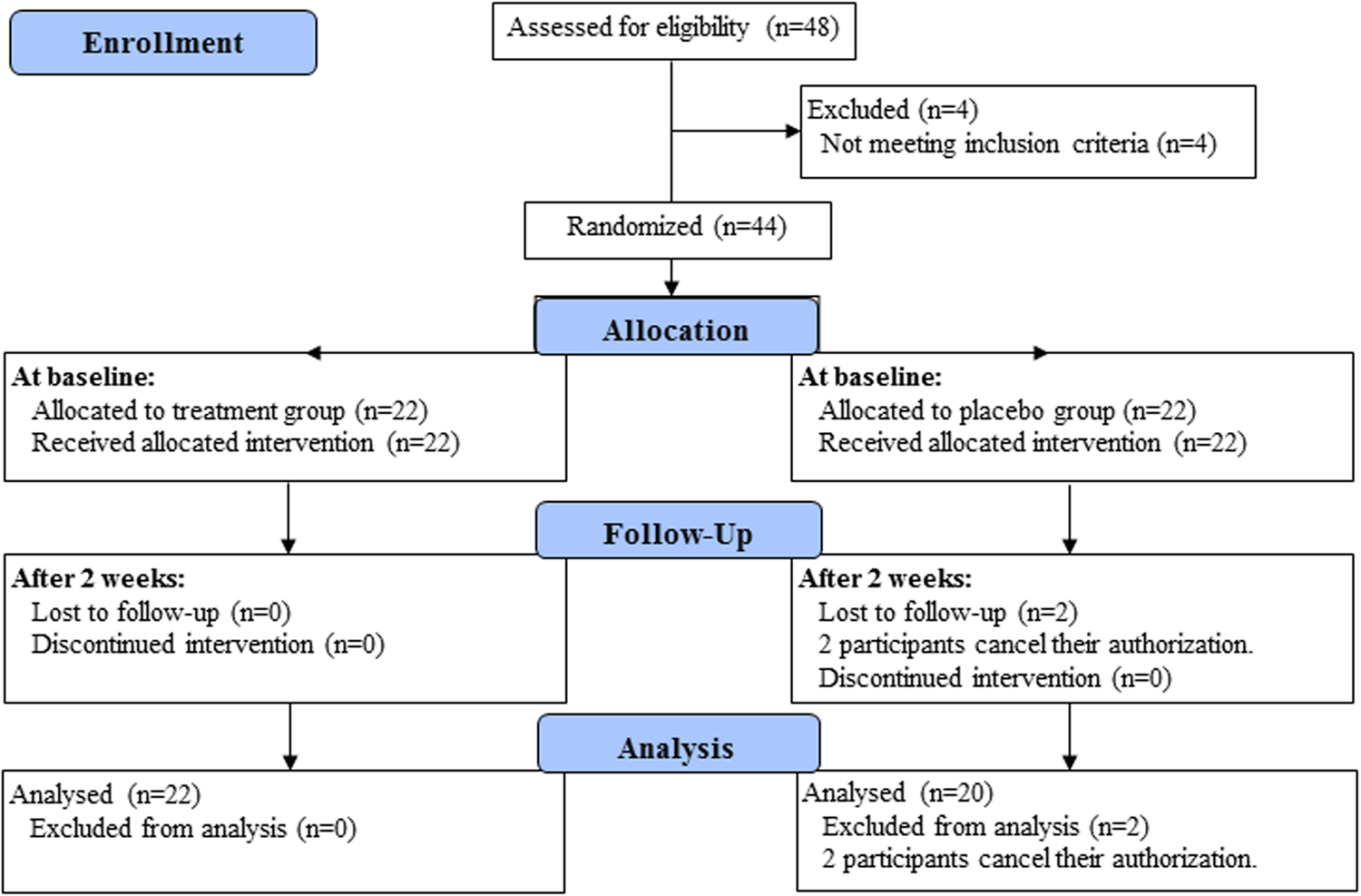
The flow chart of the study.

This clinical trial had been registered in the Chinese Clinical Trial Registry (ChiCTR) (registration number: ChiCTR-TRC-12003074, registration date: November 9, 2012), and the research protocol was approved by the Ethics Committee of Beijing Hospital, Ministry of Health (approval number: 2011010). The study period is from January 1st, 2012 to December 29st, 2012. The study was performed according to the ethical principles of the Helsinki Declaration of 1975 (and as revised in 2000). Written, informed consent was obtained from all participants or their legal guardians. This study contains the treatment and placebo groups, and none of participants or investigator was aware of treatment allocation in the treatment and placebo group until study completion. The flow chart of the study is displayed in Fig. 5. The XXMT intervention lasted 2 weeks. During the course of treatment, the placebo group was treated with aspirin, atorvastatin, symptomatic treatment, placebo tablets (oral 0.8 g for each time, 3 times per day), and supportive treatment. The treatment group was treated with the same basic treatment plus TianXinTai XXMT at the same dose. The appearance, smell and taste of the placebo tablets were designed to be identical to the treatment tablets. TianXinTai XXMT and placebo tablets were provided by Jilin Huakang Pharmaceutical Co., Ltd. Vital signs and adverse events were recorded at every visit, while the results of the laboratory tests and physical examinations were recorded at baseline and at the final visit.

44 participants were included in this study and signed the informed consent. Among those, 22 patients were randomly allocated to the XXMT treatment group and the 22 patients were allocated to the placebo control group. Gender, age, education level and ischemic lesion areas of the placebo group were matched with the treatment group. As 2 participants in placebo control group cancel their authorization, 42 participants were included into the results analysis eventually (demographic details in Table 1). To ensure the quality of the neurological assessment, investigators were trained before the start of the study. All participants received a series of neurological examinations and an fMRI scanning session that was viewed by the professional imaging staff at baseline and at the second visit (after two weeks). Participants’ inclusion criteria included the following: (1) meet acute ischemic stroke diagnosis criteria; (2) NIHSS score ≥ 3 and ≤ 10; (3) age ≥ 40 years and ≤ 80 years; and (4) patients or legal guardians provide signed informed consent forms. Exclusion criteria included the following: (1) cerebral hemorrhage or subarachnoid hemorrhage; (2) stroke caused by brain tumor or brain trauma; (3) cerebral embolism due to rheumatic heart disease, coronary artery disease and other heart cardiogenic factors; (4) associated with liver, kidney, hematopoietic system, endocrine system, bone and joint disease; (5) schizophrenia or severe dementia; and (6) undergoing cardiac pacemaker surgery, percutaneous coronary intervention and coronary artery bypass surgery which cannot be performed with MRI.

### Common stroke scale evaluation

A series of neurological assessments were performed, including the NIHSS^32^, mRS^39^, SSQOL^33^, and Barthel index^40^.

### Blood biomarker detection

In this study, blood samples from an anticoagulated peripheral vein from each patient were collected at the First Affiliated Hospital of AnHui College of Traditional Chinese Medicine. Then, we used the blood samples to measure the fibrinogen, red blood cell specific volume (HCT), total cholesterol (CHO), triglyceride (TG), low-density lipoprotein (LDL), high-density lipoprotein (HDL), and other indicators.

### MRI data acquisition

MRI data acquisition was performed using a Siemens Trio 1.5 Tesla scanner (Trio; Siemens, Erlangen, Germany) in the First Affiliated Hospital of Anhui Medical University. Foam padding and headphones were used to reduce head motion and scanner noise. T1-weighted structural images were acquired using 3-dimensional (3D) magnetization-prepared rapid gradient echo sequences: 176 sagittal slices, repetition time (TR) = 1900 ms, echo time (TE) = 3.93 ms, slice thickness = 1 mm, flip angle = 9°, field of view (FOV) = 256 mm × 256 mm and gap = 9. T2-weighted structural images were acquired using 3D magnetization-prepared rapid gradient echo sequences: 25 sagittal slices, TR = 4880 ms, TE = 116 ms, slice thickness = 5 mm, flip angle = 150°, FOV = 256 mm × 256 mm and gap = 9.

The resting-state functional images were acquired using an echo-planar imaging sequence: 200 volumes, 33 axial slices, TR = 2940 ms, TE = 45 ms, slice thickness = 3.5 mm, flip angle = 90°, FOV = 230 mm × 230 mm, and acquisition matrix = 64 × 64. The scan lasted for 600 s.

### Image data preprocessing and analysis

The infarct volume of each patient was measured manually on T1-weighted images and referenced by T2-weighted structural images by two experienced radiologists, and the average of these measures was used for statistical analysis. MRIcron software (http://www.nitrc.org/projects/mricron/) was utilized to simultaneously view and defined the anatomical regions in the coronal, axial, and sagittal planes. Next, each patient’s infarct structure was calculated as the total number of voxels in the final image. Then, the bilateral cerebral infarct lesion volume was calculated together.

The resting-state fMRI image data were preprocessed by SPM8 (http://www.fil.ion.ucl.ac.uk/spm) and Data Processing Assistant for Resting-State fMRI (DPARSF)^41^. The first 5 volumes from each subject were discarded because the participants needed time to adapt to the scanning noise and the signal needed time to reach equilibrium. The remaining 195 volumes were corrected for the acquisition time delay between different slices. Then, head motion parameters were estimated, and each volume was realigned to the mean map of the whole volume to correct for geometrical displacements using a six-parameter rigid-body transformation. Five subjects, including three in the placebo group and two in the treatment group, were excluded from further analyses because they had maximum displacements in one or more of the orthogonal directions higher than 3 mm or a maximum rotation higher than 3.0°. The data were then normalized to the standard EPI template, after which the data were re-sampled to 3 × 3 × 3 mm^3^ voxels. The normalized data were smoothed with an 8 mm full-width at half-maximum (FWHM) Gaussian kernel.

We performed ICA using the group ICA (GIFT, http://icatb.sourceforge.net/), which included three stages, data reduction, ICA application, and back-reconstruction for each subject. Firstly, the data of the participants were narrowed using principal component analysis according to the selected number of components.

Firstly, the data from each participant underwent principal component analysis to reduce the computational complexity of the analysis according to the selected number of components and most of the content of the data was preserved. Secondly, ICA was conducted to separate the data using an extended infomax algorithm. Further, to increase the stability of the estimated components, we used the Icasso algorithm (http://www.cis.hut.fi/jhimberg/icasso) (Himberg, *et al*., 2004) and repeated the ICA estimation 10 times with bootstrapping and permutation. We obtained 20 independent components (ICs), and finally the independent components and time courses for participants were constructed. Lastly, the mean spatial maps for each group were transformed into z-scores for display.

The aDMN, pDMN, lFP and rFP components were identified by visual inspection. The individual-level components were obtained from back-reconstruction and converted into z-scores. Independent component patterns representing aDMN, pDMN, lFP, rFP were identified using one-sample t-test (*P* < 0.001) in each group at baseline and follow-up. Repeated-ANOVA was used to test between-group differences.

Moreover, we also focused on the effect of XXMT on the motor network. Here, six ROIs involved in motor control and motor execution function were selected according to a study of Inman *et al*., including the bilateral primary motor area (M1), the premotor cortex (pMC), and the superior parietal cortex (SP)^37^. The regional rest-fMRI mean time series were obtained for each of the ROIs by averaging the blood-oxygen-level-dependent (BOLD) signal across all voxels within each spherical ROI. Pearson correlation coefficients between each pair of all ROIs were further computed to produce a symmetric correlation matrix for each subject. The influence of drug and time on the motor network was analyzed by using repeated-ANOVA and was presented by the Brain Net Viewer^42^.

### Statistical analysis 

#### Common stroke scale test data analysis

Statistical analyses were mainly performed in SPSS 18.0. Chi-square test was used to analyze gender difference between two groups. Then, a two-sample t-test was used to compare the differences in baseline age and education between groups. Repeated-ANOVA was used to analyze the interaction of group and time on common stroke scale scores, with age, gender, education, and baseline infarct volume as covariates.

#### Functional connectivity analysis

A full factorial design implemented in SPM was used to estimate the interactive effect between group and time on the function connectivity of the aDMN, pDMN, lFP, rFP. We identified areas that exhibited the strongest intergroup differences as ROIs and extracted the mean value for subsequent post-hoc analyses. Thresholds were set at FDR-corrected *P* = 0.05. ROI-wise analyses on motor function connectivity were then conducted among the six ROIs, which related to the motor execution function we defined earlier. Thresholds were set at FDR-corrected *P* = 0.05.

A partial correlation between stroke scale scores and functional connectivity of ROIs that represented a significant interaction effect between time and group in the aDMN, pDMN, lFP, and rFP was examined in the placebo group and the drug group using Surfstat matlab toolbox (http://www.math.mcgill.ca/keith/surfstat/), with age, gender and education level as covariates. The correlation coefficients were further transformed to Fisher’s Z scores to be compared between these two groups.

## Electronic supplementary material

Supplementary Information
