# Supplementary material for: The positive effects of Xueshuan Xinmai tablets on brain functional connectivity in acute ischemic stroke: a placebo controlled randomized trial
Source: Sci Rep. 2017 Nov 10;7:15244. doi: 10.1038/s41598-017-15456-9 (PMC5681502; doi:10.1038/s41598-017-15456-9)
Supplement: Supplementary file 1 — Supplementary Information [file 41598_2017_15456_MOESM1_ESM.pdf]

The positive effects of Xueshuan Xinmai tablets on brain functional connectivity in acute ischemic stroke: a placebo controlled randomized trial

Dongfeng Wei[1,4,#], Daojun Xie[2,#], He Li[1,4,#], Yaojing Chen[3,4], Di Qi[3,4], Yujiao Wang[5], Yangjun Zhang[3,4], Kewei Chen[4,6], Chuanfu Li[2,*] and Zhanjun Zhang[3,4,*]

[1]Institute of Basic Research in Clinical Medicine, China Academy of Chinese Medical Sciences, Beijing 100700, P. R. China

[2]The First Affiliated Hospital of Anhui University of traditional Chinese Medicine, Hefei 230031, P. R. China

[3]State Key Laboratory of Cognitive Neuroscience and Learning & IDG/McGovern Institute for Brain Research, Beijing Normal University, Beijing 100875, P. R. China

[4]BABRI Centre, Beijing Normal University, Beijing 100875, P. R. China

[5]Graduate School of Anhui University of traditional Chinese Medicine, Hefei 230038, P. R. China

[6]Banner Alzheimer's Institute, Phoenix, Arizona 85006, USA

[#] These authors contributed equally to this work.

[*] Corresponding Authors

Zhanjun Zhang, MD, Prof, State Key Laboratory of Cognitive Neuroscience and Learning & IDG/McGovern Institute for Brain Research, Beijing Normal University, Beijing 100875, China. Tel: +86 1058802005; Fax: +86 1058802005; E-mail: zhang_rzs@ bnu.edu.cn.

Chuanfu Li, MD, Prof, The First Affiliated Hospital of Anhui University of traditional

Chinese Medicine, Hefei 230031, P. R. China. Tel: +86 55162850180; Fax: +86 55162850180; E-mail: 13956078816@126.com

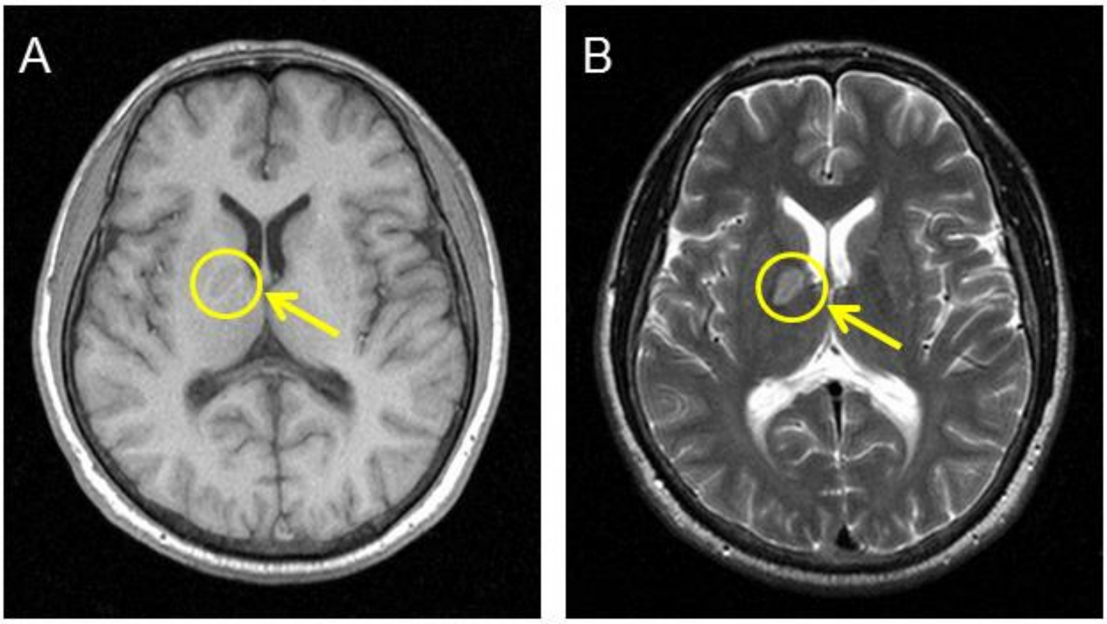

Figure S1. Example of an acute ischemic stroke patient with infarct lesion in the basal ganglion on T1 and T2 weighted MRI. (A) T1 weighted MRI. (B) T2 weighted MRI.

Table S1. Distribution of ischemic lesion regions

Brain regions	Placebo group (n=20)	Treatment group (n=22)
Total	64(100%)	66(100%)
Basal ganglia	27(42.2%)	33(50%)
Corona radiata	17(26.6%)	15(22.7%)
Brainstem	7(10.9%)	11(16.7%)
Thalamus	4(6.3)	4(6.1%)
Caudate	1(1.6%)	1(1.5%)
External capsule	1(1.6%)	1(1.5%)
Temporal lobe	3(4.7%)	1(1.5%)
Parietal lobe	1(1.6%)	-
Frontal lobe	1(1.6%)	-
Occipital lobe	2(3.1%)	-

Table S2. Correlations between functional connectivity and common stroke scale scores

Brain regions	NIHSS		SSQOL	
	r value	*P* value	r value	*P* value
Right medial frontal cortex	0.123	0.494	0.170	0.344
Left pecuneus	0.459	0.007**	0.531	0.001**
Left opercular inferior frontal cortex	0.271	0.126	0.334	0.057
Left inferior parietal gyrus	-0.171	0.341	-0.399	0.021*
Left postcentral	0.067	0.711	0.225	0.207
Right angular	0.026	0.885	-0.317	0.072
Right inferior parietal gyrus	-0.216	0.226	-0.467	0.006**

*P<0.05, **P<0.01.
